# The Neuroprotective Effects of Phenolic Acids: Molecular Mechanism of Action

**DOI:** 10.3390/nu9050477

**Published:** 2017-05-10

**Authors:** Dominik Szwajgier, Kamila Borowiec, Katarzyna Pustelniak

**Affiliations:** Department of Biotechnology, Human Nutrition and the Science of Food Commodities, University of Life Sciences in Lublin, Lublin 20704, Poland; dszwajgier@hotmail.com (D.S.); kasia.pustus@wp.pl (K.P.)

**Keywords:** cinnamic acids, benzoic acids, polyphenols, neuroprotection, neuroinflammation, central nervous system, neuron, glial cell, neurological disorder

## Abstract

The neuroprotective role of phenolic acids from food has previously been reported by many authors. In this review, the role of phenolic acids in ameliorating depression, ischemia/reperfusion injury, neuroinflammation, apoptosis, glutamate-induced toxicity, epilepsy, imbalance after traumatic brain injury, hyperinsulinemia-induced memory impairment, hearing and vision disturbances, Parkinson’s disease, Huntington’s disease, anti-amyotrophic lateral sclerosis, Chagas disease and other less distributed diseases is discussed. This review covers the in vitro, ex vivo and in vivo studies concerning the prevention and treatment of neurological disorders (on the biochemical and gene expression levels) by phenolic acids.

## 1. Introduction

**Figure 1:**
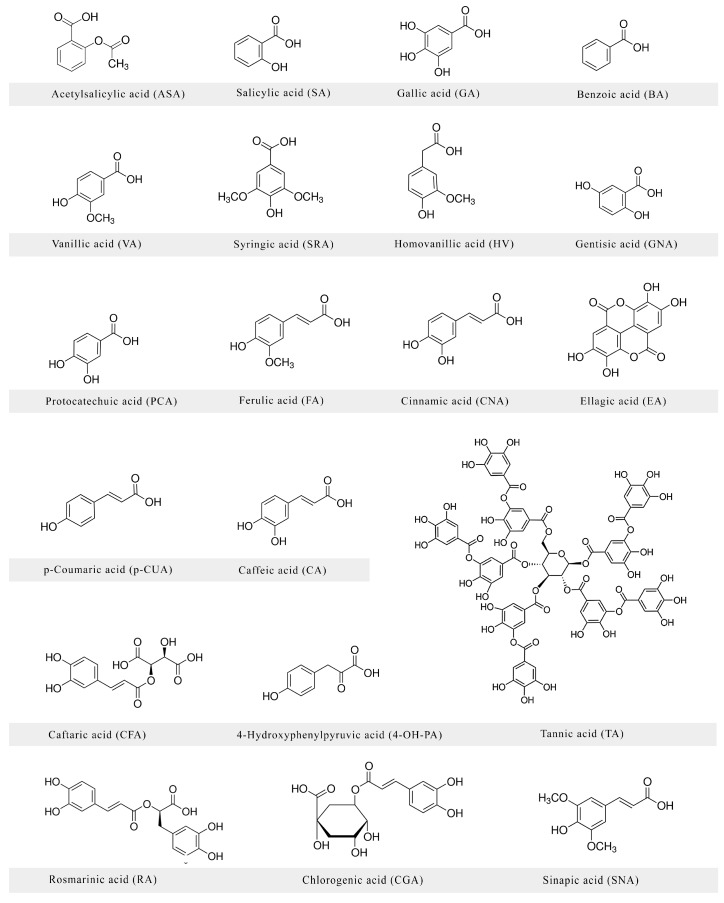
Chemical structures of phenolic acids discussed in this work.

Phenolic acids are one of the main classes of polyphenols. They are abundantly present in foods such as berries [1], nuts [2], coffee and tea [3] and whole grains [4]. Importantly, a recent meta-analysis showed that phenolic acid-rich foods decrease the risk of depression [5,6]. Figure 1 presents the chemical structures of phenolic acids discussed in this work. Previously, authors focused mainly on the antioxidant and antiradical activities of phenolic acids. However, in recent years, the interest in protecting neurons and glial cells by phenolic acids has considerably increased, and a great number of works elaborating the neuroprotective role of phenolic acids has been published. Changes in the central nervous system, as well as in the peripheral parts of the nervous system, including sense organs, affect the patient’s behavior and quality of life. Recently, we published a review paper on the anti-Alzheimer and cognition-enhancing role of phenolic acids originating from food [7]. In the following (Table 1), we present a review collating original papers concerning many other and previously omitted aspects of the neuroprotective role of phenolic acids originating from food. The presented review serves as assistance for quick access to the most prominent neuroprotective actions of phenolic acids.

## 2. Neuroprotective Activities of Phenolic Acids

## 3. Penetration of Brain by Phenolic Acids

Previously, it has been estimated that the daily consumption of phenolic acids is noticeable and totals ≈200 mg [111,112]. Moreover, the pharmacokinetic properties of phenolic acids are excellent. Bourne and Rice-Evans (1998) showed that the peak concentration of ferulic acid in plasma occurred 7–9 h after the consumption of tomatoes (360–640 g), with the recovery of the phenolic acid reaching 11–25% of the amount consumed [113]. Recently, a cross-sectional analysis of the consumption of polyphenols (involving 10 European countries and over half a million participants) revealed that the total amount of these secondary plant metabolites was high (744 mg/day in men and 584 mg/day in women in Greece to 1786 mg/day in men and 1626 mg/day in women in Denmark). Among polyphenols, phenolic acids represented the largest part (52.5–56.9% in women and men, respectively) in the diets of all groups, with the exception of men in the Mediterranean countries and “health-conscious” consumers in the United Kingdom (predominantly vegetarians). However, in the Mediterranean countries and in the “health-conscious” group in the U.K., phenolic acids were the second most distributed polyphenols (34–44%). Generally, hydroxycinnamic acids (ranging from 27% in women from the “health-conscious” group in the U.K. to 53% in men from non- Mediterranean countries) were the most important contributors to total polyphenols in the diet. The most important dietary source of phenolic acids in all studied European countries was coffee (58–75%), and the most distributed phenolic acids were caffeoylquinic acids (mainly 5-caffeoylquinic, 4-caffeoylquinic and 3-caffeoylquinic acid), followed by feruloylquinic, gallic, galloylquinic, 4-hydroxyphenylacetic, homovanillic, 3,4-dihydroxyphenylacetic and dihydro-p-coumaric acids [114]. Other major research papers have confirmed the high dietary intake of phenolic acids. Tresserra-Rimbau et al. (2013) calculated that the mean consumption of phenolic acids in a group of 7200 participants was 304 ± 156 mg/day (a parallel-group, aged 55–80 years; a validated one-year food frequency questionnaire in a multicenter, randomized, controlled five-year feeding trial). Phenolic acids were the main polyphenolics consumed (33% of all polyphenols), and 5-caffeoylquinic acid was the most abundant individual polyphenolic compound. Other phenolic acids broadly consumed were: 3-caffeoylquinic acid (49.75 ± 34.18 mg/day), 4-caffeoylquinic acid (42.60 ± 31.79 mg/day), ferulic acid (14.32 ± 14.35 mg/day), 5-feruloylquinic acid 7.24 ± 5.56 (mg/day), 4-feruloylquinic acid (6.17 ± 4.81 mg/day), syringic acid (4.82 ± 4.76 mg/day) and verbascoside (4.61 ± 7.00 mg/day) [115]. Grosso et al. (2014), in a study involving 10,477 persons aged 45–69 years (a validated 148-item food frequency questionnaire), estimated the daily intake of phenolic acids at 800 mg (521 mg/day as aglycone equivalents, 46% of total intake of polyphenols). The main phenolic acids were 5-caffeoylquinic and 4-caffeoylquinic acids (with average intake at 150 mg/day), followed by 3-caffeoylquinic acid (128.2 ± 111.6 mg/day), 5-*O*-galloylquinic acid (60.8 ± 45.4 mg/day), ferulic acid (43.9 ± 33.7 mg/day), stigmastanol ferulate (37.5 ± 22.6 mg/day), 5-feruloylquinic acid (27.9 ± 14.3 mg/day), gallic acid 25.0 ± 11.2 mg/day) and 4-feruloylquinic acid (20.4 ± 12 mg/day) [116]. 

The experimental data collated in Table 1 prove the positive role of phenolic acids in an indirect manner. The amount of phenolic acids administered to experimental animals in feed is known, but the authors did not study the content of phenolic acids in the brain. Therefore, the activity of phenolic acids in brains (on the biochemical and gene expression levels, amelioration of the enzyme activity changes) was discussed only by comparison with reference groups of animals fed with a standard diet. Although it is assumed that the transfer of polyphenols through the blood-brain barrier is limited, a considerable number of original papers confirm the presence of absorbed phenolic acid compounds in the brain. Phenolic acids can be accumulated in the brain at pharmacologically-relevant, nanomolar or micromolar concentrations, as described below. Gallic acid has been detected in trace amounts in brains (mouse model of Alzheimer’s disease) after repeated administration of grape seed polyphenolic extract for 10 days (intragastric gavage of 50, 100 and 150 mg/kg b.w.) [117]. Protocatechuic acid was detected in brain micro-dialysates (at maximal concentration of 0.09 ± 0.07 μg/mL, ≈0.58 ± 0.45 nmol/mL) 15 min–4 h after the administration of Danshen extract (*Salvia miltiorrhiza*, intragastrically, 40 mg/kg b.w.) to adult, male Sprague-Dawley rats [118]. 3-Hydroxybenzoic, benzoic and homovanillic acids were detected (at 0.43–1.06 nmol/g, 2.53–15.63 nmol/g and 1.84–2.39 nmol/g, respectively) in extracts of freeze-dried brain tissues of male Wistar rats orally fed with the grape seed proanthocyanidin extract (125, 250, 375, 1000 mg extract/kg b.w.). The levels of phenolic acids were dependent on the dose of the extract [119]. Benzoic acid was the main phenolic acid in brains of Sprague–Dawley rats that consumed wild blueberry for four and eight weeks. Other minor phenolic acids were also detected in brains, and the sum of all detected phenolic acids was 69.0 μg/g brain (which can be estimated for nanomolar concentrations, taking into consideration the molecular masses of the various phenolic acids) [120]. In another work, 3-hydroxybenzoic and 3-(3′-hydroxyphenyl) propionic acids were accumulated at μmol concentrations in perfused brain tissues of rats fed for 11 days with grape seed polyphenol extract. Both phenolic acids were shown to accumulate in brains in a dose-dependent manner. Treatment with 250 mg extract/kg b.w./day increased brain contents of 3-hydroxybenzoic and 3-hydroxyphenylpropionic acid 3.2-fold and 7.7-fold, respectively (in comparison to controls). Furthermore, hydroxybenzoic, 4-hydroxybenzoic, 3-hydroxyphenylacetic, 3,4-dihydroxyphenylacetic and 3-hydroxyphenylpropionic acids were detected in brains, but no detectable changes in the content of these phenolic acids were observed after the treatment with various doses of the extract [121]. Ferulic acid was detected in brains (2.6 μg/g of tissue, ≈13.39 nmol/mL) after the oral administration to rats (521 μmol acid/kg b.w.), and the concentration of this acid in brains was decreased only by 50% 60 min after the consumption [122]. Other works confirm the penetration of brain by ferulic acid [123], caffeic acid and caffeic acid phenethyl ester [124] and rosmarinic acid [125]. Ferulic, caffeic, rosmarinic acids and caffeic acid phenethyl ester can also protect blood-brain barrier and brain structures [126,127,128,129]. Chlorogenic acid was detected in the cerebrospinal fluid of rats that were fed with chlorogenic acid-enriched *Eucommia ulmoides* bark extract (200 and 400 mg extract/kg b.w./day, for seven days). The levels of phenolic acid were ≈0.42–0.56 ng/mL (≈0.0011–0.0015 nmol/mL) (1 h and 1.5 h after consumption, respectively) [42]. Moreover, degradation of absorbed, more complex polyphenols from foods yielding simple phenolic acids can be observed, thus increasing the levels of bioavailable phenolic acids in the brain. For example, cyanidin 3-*O*-glucopyranoside is degraded in vivo in SH-SY5Y bone marrow neuroblastoma cells, yielding protocatechuic acid [130]. Taking the above results into consideration, it can be claimed that phenolic acids can effectively accumulate in brain achieving the levels required for the pharmacological effect.

A very interesting aspect of the neuroprotective activity of phenolic acids in biological systems is the activity rather at low and not at high concentrations, as was explicitly stated by some authors. Caffeic acid dimethyl ether, when used at lower concentrations (15–50 µmol/L), was more efficient than applied at a higher dose (at 50–100 µmol/L) for the elevation of the expression of heme oxygenase-1 in cultivated astrocytes, leading to the increased concentrations of reduced glutathione in cultured cells [131]. Similarly, ferulic acid ethyl ester effectively induced heme oxygenase-1 protein expression in cultivated astrocytes at low (5 µmol/L), but not at high concentrations (15 µmol/L), along with the maximal expression of mRNA coding for heme oxygenase-1 [132]. 

## 4. Concluding Remarks

This review was designed as a compact, comprehensive, content-rich compendium of the latest reports on the role of phenolic acids in improving neurological dysfunctions by direct positive influence on neural and glial cells. Especially, the years 2014–2016 were very fruitful in terms of the very in-depth knowledge about biochemical parameters, new specific markers and gene expression modifications caused by phenolic acids, involved in the proper functioning of neural and glial cells.

In summary, it can be stated that due to a wide distribution in natural sources, a considerable daily intake, relatively high stability in foods, as well as high intestinal absorption (in comparison to more complex polyphenols) and efficient brain absorption, phenolic acids may be considered as promising compounds for the future combination therapy of neurological disorders. 

## Figures and Tables

**Table 1:** Summary of the neuroprotective activities of phenolic acids.

**Ferulic Acid**	*Antidepressant-like Effect:* reduction of the immobility in TST and FST; increased MAO levels in the hippocampus and frontal cortex, serotonin and norepinephrine levels in the hypothalamus; inhibition of MAO-A activity in the hippocampus and frontal cortex in mice (ferulic acid at 0.01, 0.1, 1 and 10 mg/kg/day, p.o.) [8] or 20 and 40 mg/kg b.w., p.o.) [9]; improvement of TST and FST scores without affecting locomotor activity; amelioration of SOD, CAT in the blood and cerebral cortex, amelioration of GPx in the cerebral cortex; decrease of thiobarbituric acid-reactive substances’ levels in the blood, hippocampus and cerebral cortex in mice [10]; reversion of the TST and FST scores, significant alleviation of CUMS-induced depressive-like behaviors in sucrose preference test and FST, significant upregulation of the BDNF, postsynaptic density protein (PSD95) and synapsin I levels in the prefrontal cortex and hippocampus in male *Imprinting Control Region* mice (ferulic acid intravenously injected, 100 mg/kg b.w.) [11]. *Protection from Ischemia/Reperfusion Injury:* promotion of EPO synthesis (increased EPO expression) in the hippocampus and the peripheral blood of male Sprague–Dawley rats after the occlusion of the right middle cerebral artery and reperfusion after 90 min [12]; downregulation of the MEK/ERK/p90RSK signaling pathway in focal cerebral ischemic injury by the prevention of middle cerebral artery occlusion-induced injury leading to decreased phosphorylation of RAF proto-oncogene serine/threonine-protein kinase, MEK1/2 (dual specificity kinase) and ERK1/2; attenuation of the injury-induced decrease in p90RSK and BAD phosphorylation levels (ferulic acid at 100 mg/kg b.w.) [13]; amelioration of neurological deficits and increased EPO expression in the hippocampus and the peripheral blood of male Sprague–Dawley rats (induced by focal cerebral ischemia provoked by occlusion of the right middle cerebral artery and reperfusion) [12]; improvement of the neuroprotective activity of puerarin and astragaloside after the transient middle cerebral artery occlusion by reducing neurological deficits, decreased infarct volume and decreased expression levels of IL-1β and neuropeptide Y (single dose of ferulic acid, 43 mg/kg b.w., p.o.) [14]. *Antinociceptive Effects:* amelioration of the descending monoaminergic system coupled with spinal β2- and 5-hydroxytryptamine 1A receptors and the downstream of δ- and mu-opioid receptors in an animal model involving CCI-induced neuropathic pain (amelioration of mechanical allodynia and thermal hyperalgesia, elevation of spinal noradrenaline and serotonin 5-hydroxytryptamine receptors, reduction of spinal MAO-A levels) (treatment with ferulic acid at 20, 40 and 80 mg/kg b.w., p.o.) [15]. *PD:* attenuation of CCI-induced neuropathic pain (in the left sciatic nerve) in rats due to increased antioxidant and anti-inflammatory activity; decreased nociceptive thresholds, thermal hyperalgesia, mechanical hyperalgesia, tactile allodynia; reduced biochemical markers: total protein, NO, lipid peroxidase, IL-1β, and IL-6 (10, 20 or 30 mg/kg b.w., p.o.) [16]; dose-dependent amelioration of 1-methyl-4-phenyl-1,2,3,6-tetrahydropyridine-induced loss of nigrostriatal dopaminergic neurons, the decrease of the Bax/Bcl2 ratio, reduction of pro-apoptotic protein Bax levels and increased expression of anti-apoptotic protein Bcl2 in PD C57BI/6 mice model (ferulic acid was given via injections for 7 days, at a dose of 20, 40 and 80 mg/kg) [17]; antioxidant and anti-inflammatory activities; rescue of dopamine neurons in substantia nigra pars compacta area and nerve terminals in the striatum; restored SOD, CAT, glutamate levels; prevented lipid oxidation; reduced the levels of ionized calcium-binding adapter molecule (Iba-1), GFAP hyperactivity, pro-inflammatory cytokines; and reduced COX-2 and iNOS activities in the studies using the rotenone-induced rat model of PD (chronic administration of ferulic acid for 4 weeks at a dose of 50 mg/kg b.w.) [18]. *Inflammation:* inhibition of the LPS-induced microglial inflammation (without cytotoxicity) by partial targeting of ERK signaling and attenuation of ERK; significant inhibition of the production of TNF-α, IL-6, IL-1β and NO; and reduction of mRNA and protein levels of COX-2 and iNOS [19]; dose-dependent prevention from LPS-induced upregulation of 3′5′-cyclic nucleotide phosphodiesterase 4B, reversion of the LPS-induced downregulation of CREB and pCREB (stimulation the cAMP/CREB signaling pathway) in PC12 cells [20]. *TBI:* attenuation of oxidative stress caused by TBI; restriction of H_2_O_2_-induced DNA fragmentation; downregulation of ROS caused by reduced mRNA gene expression; attenuation of inflammation and apoptosis; upregulation of *BDNF* gene expression; downregulation of iNOS, endothelial NOS, neuronal NOS, COX-2, IL-1β, TNF-α, SOD, as well as apoptosis-related genes (Fas-associated protein with death domain, Casp-9 and BCL-2) in Neuro-2a cells in vitro [21]. *Anti-allergic Effect:* restoration of Th1/Th2 balance by modulation of dendritic cell function; reduction of ovalbumin-specific immunoglobulin E and elevation of the immunoglobulin G2 antibody serum levels; inhibition of the production of eotaxin, Th2 cytokines (IL-4, IL-5 and IL-13) and proinflammatory cytokines; elevated production of Th1, interferon-γ in bronchoalveolar lavage fluid and the culture supernatant of spleen cells; reduced expression of proinflammatory cytokines IL-1β, IL-6 and TNF-α; increased expression of Notch ligand Delta-like 4 (DII4), MHX class II and CD40 protein; T-cell proliferation and Th1 cell polarization in dendritic cell cultures in an asthmatic mouse model with ovalbumin-induced Th2-mediated allergic asthma (animals were orally fed with ferulic acid at 25, 50 and 100 mg/kg b.w.) [22]. *Anti-apoptotic Activity:* inhibition of the p38 MAPK pathway and apoptosis by increasing the cell viability, preventing membrane damage and increasing the SOD activity; reduced intracellular free Ca^2+^ ion levels, lipid peroxidation, Casp-3 and COX-2 activation; reduced PGE2 production; increased scavenging of ROS in hypoxia-stressed PC12 cells [23]; protection against 2′-azobis(2-amidinopropane) dihydrochloride-induced oxidative stress leading to the cell survival by elevating CAT and SOD activities, mitochondrial membrane potential, reduced MDA levels, reduced LDH release from PC12 cells, and accumulation of intracellular Ca^2+^ levels in PC12 cells [24].
**Caffeic Acid**	*Inflammation:* antioxidant and anti-inflammatory activity, reduction (in a dose-dependent manner) of the cytokine levels in serum and whole brain in the LPS-induced model of inflammation in mice (caffeic acid administered orally at 30 mg/kg b.w.) [25]. *PD:* inhibition (in a dose-dependent manner) of α-synuclein fibrillation in the presence of escitalopram [26]. *Glutamate-induced Toxicity:* in vitro neuroprotection activity of SH-SY5Y cells by caffeic acid derivatives from *Arctium lappa* roots: 1,5-*O*-dicaffeoyl-3-*O*-(4-malic acid methyl ester)-quinic acid, 3,5-*O*-dicaffeoyl-quinic acid methyl ester, 3,4-*O*-dicaffeoyl-quinic acid methyl ester, 4,5-*O*-dicaffeoyl-quinic acid methyl ester, (2E)-1,4-dimethyl-2-[(4-hydroxyphenyl)methyl]-2-butenedioic acid, chlorogenic acid methyl ester, caffeic acid methyl ester, 3,4,3′,4′-tetrahydroxy-δ-truxinate [27]. *Anti-epileptic Activity:* reduction of the levels of free radicals and DNA damage in the kindling CF-1 male mice model of epilepsy induced by PTZ (caffeic acid at 1, 4 or 8 mg/kg b.w., i.p.) [28]; reduction of the latency to sleep in the diazepam-induced sleeping time test, decreased pilocarpine-induced genotoxic damage in acute seizure models in mice (caffeic acid at 4 or 8 mg/kg b.w., i.p.) [29]. *Memory Impairment in Hyperinsulinemia:* high-fat diet-induced hyperinsulinemic rats: amelioration of glucose uptake and cell viability, improvement of memory impairment and brain glucose metabolism via significant reduction of plasma glucose and insulin levels, amelioration of the cerebral insulin and leptin signaling pathways (insulin receptor, phosphatidylinositol-3-kinase, protein kinase B, and insulin-degrading enzyme, leptin receptor and phosphorylated Janus tyrosine kinase 2 Tyr813/Janus tyrosine kinase 2 in the cortex of rats) (caffeic acid at 30 mg/kg b.w., p.o.) [30].
**Caffeic Acid Phenethyl Ester**	*Antioxidant Activity:* protection of brain tissue against ionizing radiation-induced oxidative damage by amelioration of the SOD activity in brains of male albino Sprague–Dawley rats (10 µmol ester/kg b.w./day, i.p., for 10 days after irradiation) [31]; amelioration of the redox-balancing activity, positive modulation of the transcription-factor, stimulation of Nrf2, inhibition of NF-κB activity, as well as signal transducer and activator of transcription 3 (STAT3) [32]; attenuation of the ifosfamide-induced central neurotoxicity in Wistar rats (after intraperitoneal injection) by attenuating the increase in MDA and protein carbonyl content in brain tissue (10 µmol ester/kg b.w., i.p.) [33,34]. *Anti-apoptotic Activity:* protection of PC12 cells from the cellular death induced by neurotoxin methyl-4-phenylpyridinium by increasing the neurite network (promotion of the formation, elongation and ramification; inhibition of the shortage of neurites); increasing the expression of neuron-typical proteins responsible for axonal growth (growth-associated protein 43) and synaptogenesis (synaptophysin and synapsin I) [35]; reduction of the incidents of volatile anesthetic sevoflurane-induced neurodegeneration (neurotoxicity in neonatal rats) and apoptosis by activation of the phosphatidylinositide 3-kinase/protein kinase B signaling pathway, downregulation of the expression of Bax and BAD, upregulation of Bcl-2 and Bcl-extra-large levels and modification of the expression of MAPK levels (rat pups were administered with ester at 10, 20 or 40 mg/kg b.w. from postnatal Days 1–15) [36]. *Inflammation:* significant inhibition of the expressions of NOS, COX-2 and the production of NO; increased expression of heme oxygenase-1 and EPO in microglia in in vitro tests [37]. *Anticancer Activity:* reduction of NO, intracellular Ca^2+^ levels, and CAT activity in C6 glioma cells when combined with Dasatinib (Bcr-abl tyrosine kinase inhibitor), in comparison to Dasatinib applied alone [38]. *Huntington Disease:* reduction of striatal damage, immunoreactivity to glial GFAP and lymphocyte common antigen (CD45) (markers of astrocyte and microglia activation); reduced behavioral deficits tested on the rotarod in the chemical model of Huntington disease (male C57BL/6 mice); reduced mortality of cultured striatal neurons of male C57BL/6 mice after the induction of the inflammation by 3NP [39].
**Chlorogenic Acid**	*Reversing of the Glutamate-induced Toxicity:* inhibition of the glutamate-induced increase of intracellular Ca^2+^ concentrations, as well as glutamate-induced death of primary cells isolated from mouse cortical neurons (cerebral cortex) [40]. *Inflammation:* attenuation of herpes simplex virus-1-induced inflammation in BV2 microglia, improving cell viability and increasing (at the mRNA and protein levels) Toll-like receptor 2, Toll-like receptor 9 and myeloid differentiation factor 88; significant inhibition of mRNA concentration, NF-κB p65 expression and TNF-α and IL-6 levels in microglia [41]. *Antidepressant Effect:* stimulation of axon and dendrite growth, promotion of serotonin release through enhancing synapsin I expression (via 5-hydroxytryptamine receptors) in the cells of fetal raphe neurons in vitro [42]. *Antioxidant Activity:* amelioration of the decrease of MDA and ROS levels in rat cortical slices after the H_2_O_2_-induced oxidative stress [43]. *Anti-epileptic:* reduction of the pilocarpine-induced epilepsy (seizures) in mice by reducing the lipid peroxidation and nitrite content, as well as the mRNA expressions of *N*-methyl-d-aspartate receptor, metabotropic glutamate receptor 1 and metabotropic glutamate receptor 5 (chlorogenic acid administered at 5 mg/kg b.w., p.o.) [44]. *Anti-apoptotic:* dose-dependent increase of cell viability, cell distribution ratio at the G2/M and S phases; promotion of cell differentiation by preventing ethanol-induced apoptosis in rat PC12 cells by enhancing the expression of growth-associated protein-43 (GAP-43); inhibition of the mitochondrial apoptotic pathway by promoting mitochondria transmembrane potential, upregulation of the expression of Bcl-2 and downregulation of the expression of Casp-3 [45].
**Chlorogenic Acid and Its Metabolites**	*Reversing of the Glutamate-induced Toxicity:* protection from nitroprusside-induced NO generation (chlorogenic and caffeic acids), significant reduction of excitotoxicity (ferulic and caffeic acids), protection against H_2_O_2_-induced proteasome inhibition and caspase-dependent intrinsic apoptosis, as well as endoplasmic reticulum stress (caffeic acid) in primary cultures of rat cerebellar granule neurons [46].
**Rosmarinic Acid**	*Antioxidant Activity:* induction/activation of the nuclear factor erythroid 2-related factor 2-antioxidant-responsive element (Nrf2-ARE) signaling pathway and potentiation of the Nrf2/HO-1 signaling pathway leading to the enhanced endogenous antioxidant defense (decreased superoxide production, reduced expression of 4-hydroxynonenal and upregulation of SOD) in a rat model of noise-induced superoxide production and overexpression of the lipid peroxidation marker 4-hydroxynonenal (rosmarinic acid administered at 10 mg/kg b.w., i.p.) [47]; protection against the iron-induced neurotoxicity in neuroblastoma SK-N-SH cells [48]; prevention of the progression of oxidative stress caused by H_2_O_2_ in C6 glial cells by increasing the cell viability and inhibiting the cellular lipid peroxidation, reduction of H_2_O_2_-induced expression of inducible iNOS and COX-2 at the transcriptional level, downregulation of iNOS and COX-2 protein expression in H_2_O_2_-induced C6 glial cells [49]; antioxidant effect, inhibition of MAO-A and MAO-B and catechol-*O*-methyl transferase (COMT) with no cytotoxicity on polymorphonuclear rat cells [50]; enhancing the antioxidant status, decreasing the oxidative stress, efficiently ameliorating inflammatory mechanisms by downregulation of NF-κB and pro-inflammatory cytokines after spinal cord injury in Wistar rats (rosmarinic acid administered at 10 mg/kg b.w., i.p.) [51]. *Anti-epileptic Activity:* increasing the latency and decreasing the percentage of seizure incidents, reducing the levels of free radicals and DNA damage in the kindling CF-1 male mice model of epilepsy induced by PTZ (rosmarinic acid at 1, 2 or 4 mg/kg b.w., i.p.) [28]; attenuation of seizures, mitigation of the oxidative stress, augmentation of the activity of defensive systems, reduction of MDA and nitrite content and increase of CAT activity; prevention of the hippocampal neuronal loss in CA1 and CA3 regions and mossy fiber sprouting in the kainite model of temporal lobe epilepsy in rats (rosmarinic acid administered at 10 mg/kg b.w./d, i.p.) [52]; acute anticonvulsant-like activity against seizures via increased latency to myoclonic jerks and generalized seizure durations in the C57BL/6 female mouse model with PTZ-induced epilepsy (rosmarinic acid at 3 or 30 mg/kg b.w., p.o., for 14 days) [53]; improvement (in combination with diazepam) of the latency to first seizures, reduction of the latency to sleep in the diazepam-induced sleeping time test in a model of PTZ-induced seizures in mice, decreased pilocarpine-induced genotoxic damage in a mice acute seizure model (rosmarinic acid at 2 or 4 mg/kg b.w., i.p.) [29]. *Huntington Disease* improvement of the behavioral abnormalities and attenuation of the oxidative stress in 3NP-treated rats (an animal model of Huntington disease) (rosmarinic acid at 12 mg/kg b.w., nasal delivery) [54]. *Antidepressant Effect:* downregulation of mitogen-activated protein kinase phosphatase-1, upregulation of BDNF and modulation of dopamine and corticosterone synthesis in TST in a model of depression in mice with bupropion as a positive control (rosmarinic acid administered for 7 days at 5 and 10 mg/kg b.w./day) [55]. *Anti-tauopathy Activity:* counteracting the stress-induced tauopathy by efficient suppression of the elevation of P-tau and insoluble P-tau formation, reversion of the abnormal changes of chaperones and peptidyl-prolyl cis/trans isomerase (Pin1) in middle-aged mice with induced chronic restraint stress [56]. *Long-term Potentiation:* enhancement of baseline field excitatory postsynaptic potentials (fEPSPs) following high-frequency stimulation in CA1 synapses, increase of the expression of BDNF and ionotropic AMPA glutamate receptor 2 (GluR-2) proteins and prevention of cell death in scopolamine-exposed organotypic hippocampal slice cultures [57]. *Anticancer Activity:* dose-dependent suppression of cell proliferation and cytotoxic effect on glioblastoma cells without antioxidant effect (at 171.3–290.5 μmol/L), but at higher doses, a prooxidant effect was observed, leading to cell death through necrosis [58]. *Inflammation:* decrease of COX-2, PGE-2, IL-1β, matrix metallopeptidase 2 and NO levels in male Wistar rats that underwent CCI (rosmarinic acid at 40 mg/kg b.w., i.p., after 7 and 14 days) [59].
**P-Coumaric Acid**	*Protection from Ischemia/ Reperfusion Injury:* decrease of MDA, increase of NRF1 levels and SOD activity, reduction of ischemic fiber degeneration and Aβ protein expressions in rats’ sciatic nerve segments after abdominal aorta clamping (p-coumaric acid at 100 mg phenolic acid/kg b.w.) [60]; decrease of the oxidative damage, focal ischemia and neurological deficit scores in rat brains subjected to cerebral ischemia (via intraluminal monofilament occlusion model) due to the antioxidant and antiapoptotic activity (p-coumaric acid at 100 mg phenolic acid/kg b.w.) [61]; decrease of MDA, hypoxia-inducible factor-1α levels and NF-κB immunopositive neuron number; increase of NRF1, SOD activity and the number of normal neurons after ischemia-reperfusion injury of the spinal cord (via infrarenal aorta cross-clamping model) in rats (p-coumaric acid at 100 mg/kg b.w.) [62]. *Anticancer Activity:* cytotoxic effect on neuroblastoma N2a cells by generation of ROS leading to dysfunction of mitochondrial membrane, the release of cytochrome c, decreased intracellular reduced glutathione, p53-mediated upregulated accumulation of Casp-8 messenger RNA, accumulation of microtubule-associated 1A/1B light chain 3B protein (LC3-II) leading to apoptosis and autophagy [63].
**Sinapic Acid**	*PD:* partial amelioration of negative phenomena in the 6-OHDA-induced hemi-parkinsonian rat: Improved turning behavior, prevented loss of dopaminergic neurons in substantia nigra pars compacta, lowered iron reactivity and attenuated MDA and nitrite levels in midbrain homogenate (rats pretreated p.o. with sinapic acid at 20 mg/kg b.w.) [64].
**Cinnamic Aldehyde**	*Inflammation and Cognition:* reduction of COX-2 protein activity and PGE2 concentrations in frontal cortex and hippocampus; reversal of selected abnormalities (exploratory behavior, central ambulation and total ambulation-anxiety behavior, rearing, grooming, immobility period) studied in open field exploratory behavior test in mid-aged rats after the exposure to CUMS (cinnamic aldehyde at 45 and 90 mg/kg b.w., p.o., for 21 days) [65].
**Salicylic Acid**	*Antioxidant Activity:* sodium salicylate: amelioration of negative alterations in methamphetamine-induced mouse model, including scavenging of ROS, reversing of the mitochondrial dysfunction and movement abnormalities, and amelioration of the complex-I activity decrease leading to striatal dopamine depletion (sodium salicylate at 50 and 100 mg/kg b.w.) [66]; ex vivo neuroprotective and antioxidant effect in primary cortex neurons isolated from Sprague–Dawley rat brains after the oxygen stress caused by paclitaxel and cisplatin [67].
**Acetylsalicylic Acid**	*Inflammation and Antioxidant Activity:* counteracting the decrease of degenerative changes, decrease of inflammatory reactivity, and the expression of estrogen receptors (atrophy) in hippocampus caused by 2,3,7,8-tetrachlorodibenzo-p-dioxin (acetylsalicylic acid at 50 mg/kg b.w., p.o., for 21 days) [68]; reduction of the neuroinflammation markers and oxidative stress markers PGE2, 15-epilipoxin A4, 8-isoprostane and leukotriene B4 concentrations in HIV-1 transgenic rat model associated with neurocognitive disorders (acetylsalicylic acid at 10 mg/kg/day in drinking water, for 42 days) [69]. *Protection from Ischemia/ Reperfusion Injury:* reduction of the early neurological deterioration in patients with acute ischemic stroke (in combination with clopidogrel), in comparison to monotherapy (clopidogrel alone), in studies involving 690 patients aged > 40 years with minor stroke or transient ischemic attack (aspirin at 100 mg/day in combination with clopidogrel at 75 mg/day, in comparison to monotherapy with aspirin alone at 300 mg/day) [70]; significant reduction of platelet aggregation and platelet-leukocyte aggregate numbers in patients after acute ischemic stroke (1124 patients, among which 270 experienced neurological deterioration), lower incidence of neurological deterioration in patients with pre-stroke concomitant treatment with phenolic acid (acid at 200 mg/day, p.o.) [71]. *Anti-amyotrophic Lateral Sclerosis (ALS) Effect:* consumption was independently inversely associated with ALS risk, predominately in patients older than 55 years, as observed in studies involving 729 patients with newly diagnosed ALS and 7390 sex-, age-, residence- and insurance premium-matched controls [72]. *Normalization of Brain Function:* moderate enhancement of rapamycin-mediated inhibition of dendritic cells’ allostimulatory capacity: reduction of the number of mouse bone marrow-derived immature dendritic cells expressing CD40 protein and major histocompatibility complex class II (MHC II) molecules after the stimulation by LPS [73]. *Inflammation:* reduction of iron content in microglial cells by regulating the expression of iron transport proteins: downregulation of transferring receptor 1, upregulation of ferroportin 1 and ferritin expressions in microglial cells, partial reversion of LPS-induced disruption of cell iron balance under in vitro inflammatory conditions by decrease of ferritin, IL-6, TNF-α, hepcidin mRNA contents previously increased by LPS alone [74]. *Chagas Disease:* protection of the esophageal myenteric neurons from the atrophy caused by *Trypanosoma cruzi* without alterations in the esophageal wall and the myenteric neurons in infected mice [75]. *TBI:* upregulation of proteins involved in the neuroprotection of cellular pathways in Sprague–Dawley rats sustaining TBI, leading to the amelioration of previously provoked alterations in proteome and glycoproteins (acid at 30 mg/kg, i.p.) [76]. *Prevention of Hearing Loss:* decrease of the progression of the age-related hearing loss, positive retinal microvascular changes, amelioration of the mean pure tone average hearing threshold (decibels) in the better ear in studies involving 1262 Australians aged over 70 years with normal cognitive functions after 3 years of phenolic acid consumption (enteric-coated aspirin at a dose of 100 mg, p.o.) [77].
**Protocatechuic Acid**	*Antioxidant Activity:* attenuation of the loss of neurons in zebrafish and mice treated by 6-hydroxydopamine; increased cell viability, Nrf2-related factor 2 protein expression, upregulation of the expression of antioxidant enzymes such as heme oxygenase-1, SOD, CAT; decrease of MDA, NF-κB, and iNOS levels; decrease of LDH release from cells in 6-OHDA-treated PC12 cells (protocatechuic acid in combination with chrysin) [78]; protection of brain mitochondrial functions (glycemic control, reduction of oxidative stress markers) in the heart of streptozotocin-induced diabetic rats (protocatechuic acid at 50 and 100 mg/kg, p.o., for 12 weeks) [79]. *Cell Proliferation:* induction of proliferation of RSC96 Schwann cells by phosphorylation of the insulin-like growth factor-I-mediated phosphatidylinositol 3 kinase/serine-threonine kinase (IGF-IR-PI3K-Akt) pathway; activation of expression of cell nuclear antigen in a dose-dependent manner; positive modulation of expressions of cell cycle proteins cyclin D1, E and A and a knockdown of PI3K by small interfering RNA and inhibition of IGF-IR [80]; prevention of the reduction of mitochondrial membrane potential along with the increased cell viability, ameliorated mitochondrial complex I activity, reduction of the release of LDH and ROS from cells in midbrain dopaminergic neurons injured by 1-methyl-4-phenylpyridinium in Kun Ming mice [81]. *Inflammation:* inhibition of Toll-like receptor 4-mediated NF-κB and MAPKs signaling pathways and the inhibition of the LPS-induced production of TNF-α, IL-6 IL-1β and PGE2 in LPS-induced BV2 (C57BL/6) microglia [82]. *PD:* increase of tyrosine hydroxylase and dopamine receptor D2 and decrease of iNOS expression in striatum and midbrain of C57BL mice after the induction of PD by 1-methyl-4-phenyl-1,2,3,6-tetrahydropyridine (protocatechuic acid at a dose of 10 mg/kg in combination with Madopar at 125 mg/kg, i.p., for 7 days) [83]. *Inflammation:* induction of the expressions of MAPK (ERK1/2, JNK and p38) followed by the activation of downstream expressions of matrix-degrading proteolytic enzymes Pas, matrix metallopeptidase 2, and matrix metallopeptidase 9 in RSC96 Schwann cells, which modified the cell migration and the regeneration of damaged peripheral nerve [84].
**Gallic Acid**	*Antioxidant Activity:* amelioration of the intracerebroventricular streptozotocin-induced oxidative damage by normalization of thiobarbituric acid-reactive substances and total thiol contents, as well as GPx, CAT and SOD activities in the rat striatum (gallic acid at 30 mg/kg, p.o., for 26 days) [85]; amelioration of antioxidative enzymes in the development of depression (gallic acid at 0.8, 2 and 4 mg/kg b.w., p.o., for 10 days) [86]. *Traumatic Nerve Injury:* dose-dependent improvement during the peripheral nerve degeneration (amelioration of the motor coordination and sciatic nerve crush velocity) in rats with sciatic nerve crush (gallic acid at 200 mg/kg/2 mL, p.o.) [87]. *Antidepressant Effect:* amelioration of the anxiety and depression (tested in TST, elevated plus maze and novelty suppressed feeding test), reduction of the cell densities in the CA1, CA2, CA3 and DG hippocampal subdivisions after the administration of trimethyltin to Sprague–Dawley rats (gallic acid at 150 mg/kg b.w., i.p., for 14 days) [88]. *Cytotoxicity:* reversion of the cyclophosphamide-induced neurotoxicity in Wistar rats by restoration of normal levels of cerebellar and cerebral CAT, SOD, MDA, glutathione S-transferase, H_2_O_2_, GPx and nitrite levels (gallic acid at 60 and 120 mg/kg b.w., p.o., for 10 days) [89]. *Anticancer Activity:* dose-dependent cytotoxicity in DBTRG-05MG human brain glioblastoma cells by the elevation of intracellular Ca^2+^ levels in cells, increase of intracellular Ca^2+^ levels in combination with thapsigargin, increase of ROS production and activation of mitochondrial apoptotic pathways [90].
**Tannic Acid**	*Antioxidant Activity:* increase of the concentrations of NR2A and NR2B subunits of *N*-methyl-d-aspartate receptors, elevation of the activities of antioxidant enzymes, decrease of lipid peroxidation in the brain hippocampus in Wistar rats after 16-weeks exposure of animals to Al^3+^ and Pb^2+^ (tannic acid at 50 mg/kg b.w./day; a nasogastric probe was used) [91]; counteracting against Pb^2+^-induced neurochemical perturbations in Wistar rats including the reduction of oxygen radical species levels and enzymatic oxidants; amelioration of the activity of non-enzymatic antioxidants, neurotoxicity biomarkers and histological changes (tannic acid at 50 mg/kg b.w., three times a week, for two weeks) [92]. *Protection from Ischemia/reperfusion Injury:* reduction of ROS and MDA levels, elevation of SOD and NRF1 levels in brain tissues in rats with brain ischemia after middle cerebral artery occlusion induced by ethanol given intraperitoneally (tannic acid at a dose of 10 mg/kg b.w. dissolved in 10% ethanol administered within half an hour intraperitoneally) [93]; reduction of infarct size, improved neurological function, suppressed neuronal loss, downregulation of the GFAP expression, reduction of thiobarbituric acid reactive species and cytokine levels in Wistar rats after the middle cerebral artery occlusion followed by reperfusion (tannic acid at 50 mg/kg, i.p.) [94].
**Homovanillic Acid**	*Antidepressant Effect:* reduction of depressive symptoms in a 4-week, double-blind, randomized, placebo-controlled study involving 22 men and 25 women, due to the improvement of the peripheral dopaminergic activity and increased (by 11.5%) homovanillic acid concentration in plasma of overweight or obese patients with depressive symptoms (after the co-supplementation with 1.4 g cocoa extract/day corresponding to 645 mg total polyphenols/day) [95]; the lower number of suicide incidents in patients with schizophrenia and elevated homovanillic acid levels in cerebrospinal fluid (28-year follow-up studies) [96]. *Psychotic Disorders:* normalization of the disturbed dopaminergic activity in patients with psychotic spectrum disorders, especially schizophrenia, by partially taking over the functions in dopaminergic metabolism in the central nervous system [97].
**Syringic Acid**	*Protection from Ischemia/reperfusion Injury:* elevation of SOD activity, NRF-1 levels; reduced MDA, Casp-3 and Casp-9 levels leading to the reduced oxidative stress and neuronal degeneration in Sprague–Dawley rat brain tissues after cerebral ischemia caused by artery occlusion (syringic acid at 10 mg/kg b.w., i.p.) [98]; reduction of the oxidative stress and neuronal degeneration by reduction of the number of apoptotic neurons, beclin-1 protein and Casp-3-immunopositive neurons in spinal cords of Sprague–Dawley rats with spinal cord ischemia (infrarenal aortic cross-clamping model) (syringic acid at 10 mg/kg b.w., i.p.) [99]. *Vision:* prevention of retinal ganglion cells RGC-5 from H_2_O_2_-induced apoptosis through the activation of phosphatidylinositol 3-kinase/protein kinase B signaling pathway, elevated expression of the Bcl-2 regulator proteins, decrease of the expression of Bax and cleaved Casp-3 protein [100]. *Protection during Oxygen Deprivation/Reperfusion Injury:* attenuation of the injury of primary hippocampal neuronal cells by the decrease of the following: LDH leakage from cells, Bax and Casp-3 expressions, the levels of intracellular MDA, ROS, and Ca^2+^; inhibition of oxygen deprivation/reperfusion-induced increase in phosphorylated JNK and p-p38 expression; increased cell viability, restoring the intracellular SOD, mitochondrial membrane potential, and Bcl-2 expression [101].
**Ellagic Acid**	*Anticancer Activity:* reduction of the number of human neuroblastoma SH-SY5Y cells by alterations of the mitochondrial membrane potential, activation of Casp-3, Casp-9, fragmentation of DNA, and dose- and time-dependent cell apoptosis by the mitochondrial pathway [102,103]; decrease of cell proliferation, cell viability, decrease of the proportion at G0/G1 phase of the cell cycle together with increased cell population at S phase; upregulation of Death receptor 4, Death receptor 5, and MAP kinases (JNK, ERK1/2, and p38), as well as CCAAT-enhancer-binding homologous protein (CHOP) and glucose-regulated protein 78 (GRP78) expressions leading to the severe apoptosis in U251 human glioblastoma cells [104]. *PD:* restoration of the locomotion, reduction of the levels of neuroinflammatory biomarkers TNF-α and IL-1β in the striatum and in hippocampus of a rat model of PD induced by 6-OHDA (right medial forebrain bundle-lesioned rats) (ellagic acid at 50 mg/kg b.w./2 mL, by gavages) [105]; amelioration of the rotenone-induced locomotor impairment in zebrafish (adult zebrafish exposed to ellagic acid at 20 or 40 mg/kg b.w. in combination with curcumin at 20 or 40 mg/kg b.w., i.m., for 14 days) and *Drosophila melanogaster* (adult wild-Type flies exposed to ellagic acid at 0.05% and 0.1% in combination with curcumin at 0.05% and 0.1%, in feed for 7 days) (swimming behavior and poorer climbing capability, respectively) [106]. *Amnesia:* reversion of the scopolamine-induced amnesia verified in the elevated plus maze and passive avoidance paradigm tests, improvement of amnesia caused by diazepam in rats (ellagic acid at 30 or 100 mg/kg b.w., i.p.) [107]. *Inflammation:* downregulation of the p38 mitogen-activated protein kinase (p38 MAPK), amelioration of the inflammatory pain including acetic acid-induced nociception, formalin-induced nociception, and paclitaxel-induced neuropathic pain in the murine model (ellagic acid at 50 mg/kg b.w./2 mL of saline, administered as a bolus into the subcutis for 5 days) [108]. *Protection from the Ischemic Injury:* reduction of the infarct size, weight, and volume of the brain; reduced apoptosis by reduced levels of caspases, apoptotic pathway proteins, MAPK proteins, and inflammatory mediators NF-κB (p65) and p-IK-Ba in hypoxic-ischemic brains of rat pups (ellagic acid at 10, 20 or 40 mg/kg b.w., p.o.) [109]. *Protection during Oxygen* *Deprivation/Reperfusion Injury:* improvement of the rats’ nerve-related abilities, remedied infarct volumes and morphological changes in the brain enhanced content of nestin protein in the brain semi-darkness zone in a photothrombosis-induced model of brain injury in rats; elevation of β-catenin expression and *cyclin D1* gene expression in an oxygen-glucose deprivation and reperfusion model established in in vitro primary cultured neural stem cells [110].

## Abbreviations

3NP	3-Nitropropionic acid
6-OHDA	6-Hydroxydopamine
BAD	Bcl-2-associated death promoter
Bax	Bcl-2-like protein 4
BCL	B-cell lymphoma
BCL-2	B-cell lymphoma 2
Bcr-Abl	fusion between break point cluster (Bcr) gene and the Abelson (Abl) tyrosine kinase gene
BDNF	Brain-derived neurotrophic factor
b.w.	Body weight
Casp-3	Caspase-3
Casp-8	Caspase-8
Casp-9	Caspase-9
CAT	Catalase
CCI	Chronic constriction injury
CD40	Cluster of differentiation 40
COX-2	Cyclooxygenase-2
CREB	cAMP response element-binding protein
CUMS	Chronic unexpected mild stress
EPO	Erythropoietin
ERK	Extracellular signal-regulated kinase, protein-serine/threonine kinase
ERK1	Extracellular signal-regulated kinase 1, protein-serine/threonine kinase 1
ERK2	Extracellular signal-regulated kinase 2 protein-serine/threonine kinase 2
FST	Forced swimming test
GFAP	Glial fibrillary acidic protein
GPx	Glutathione peroxidase
i.p.	Intraperitoneally
IL-1β	Interleukin 1β
IL-4	Interleukin 4
IL-5	Interleukin 5
IL-6	Interleukin 6
IL-13	Interleukin 13
iNOS	Inducible nitric oxide synthase
JNK	c-Jun N-terminal kinase
LDH	Lactate dehydrogenase
LPS	Lipopolysaccharide
MAO	Monoamine oxidase
MDA	Malondialdehyde
MEK	Mitogen-activated protein kinase
MHX	major histocompatibility complex II molecules
NF-κB	Nuclear factor-κB
NO	Nitric oxide
NRF1	Nuclear respiratory factor 1
Nrf2	Nuclear factor (erythroid-derived 2)-like 2
Nrf2/HO-1	Nuclear factor (erythroid-derived 2)-like 2/heme oxygenase-1
p38	MAP Kinase (MAPK), CSBP Cytokinin-Specific Binding Protein or RK
p90RSK	MAPK-activated protein kinase-1 (MAPKAP-K1)
pCREB	phosphorylated cAMP response element-binding protein
PD	Parkinson disease
PGE2	Prostaglandin E2
p.o.	Orally
p-p38	phosphorylated p38
PTZ	Pentylenetetrazol
ROS	Reactive oxygen species
SOD	Superoxide dismutase
TBI	Traumatic brain injury
Th1	T helper type 1 (Th1) cells
Th2	T helper type 2 (Th2) cells
TNF-α	Tumor necrosis factor-α
TST	Tail suspension test
